# Photo-refractoriness reflects bet-hedging strategies deployed in unpredictable environments in male Brandt’s voles

**DOI:** 10.1186/s40851-025-00251-6

**Published:** 2025-04-28

**Authors:** Lewen Wang, Yaqi Ying, Ying Song, Ning Li, Xiao-Hui Liu, Dawei Wang

**Affiliations:** 1 State Key Laboratory for Biology of Plant Diseases and Insect Pests, Institute of Plant Protection, Chinese Academy of Agricultural Sciences, No. 2 Yuanmingyuan West Road, Haidian District, Beijing, 100193 China; 2 Western Agricultural Research Center, Chinese Academy of Agricultural Sciences, Changji, China; 3 Institute of Grassland Research, Chinese Academy of Agricultural Sciences, Key Laboratory of Biohazard Monitoring and Green Prevention and Control in Artificial Grassland, Ministry of Agriculture and Rural Affairs, Hohhot, China

**Keywords:** Photoperiodism, Brandt’s vole, Testicular development, Photo-refractoriness, Bet-hedging

## Abstract

Day length, also known as photoperiod, is an important reproductive regulatory factor in most seasonal breeders. Brandt’s vole, a long-day breeder, exhibits significant differentces in reproductive development depending on the photoperiod of the season of birth, as is seen in other rodent seasonal breeders. However, there is a lack of comprehensive studies on the effects of photoperiod across different seasons. In the present study, we investigated the impact of long (LP) and short photoperiod (SP) on postnatal development in male voles. We measured somatic and testicular parameters from weaning at three postnatal weeks (PNW3) to PNW19, weighed testis mass from birth, and confirmed the status of testicular development by observing the histological features of the seminiferous epithelium. The results showed no difference in testis mass between LP and SP males up to PNW3, with normal initiation of intratubular meiosis and the presence of leptotene/zygotene spermatocytes in both groups. From PNW4 to PNW10, SP males displayed slower growth in both somatic and testicular parameters and showed suppressed development of primary spermatocytes and Leydig cells compared to LP males. After PNW10, both groups experienced photo-refractoriness, characterized by a reversal of gonadal activity. During this stage, SP voles spontaneously initiated gonadal development and resumed the meiotic process, while LP males showed testicular degeneration accompanied by a progressive loss of germ cells ranging from spermatids to primary spermatocytes. Until PNW19, both groups reached similar testis size and mass. Interestingly, this refractoriness was observed in only half of the males in each group, suggesting a bet-hedging survival strategy that allows populations to cope with unpredictable environmental changes, such as fluctuations in temperature and food. These findings highlight the importance of photoperiod as a key environmental factor in influencing sexual maturation in young Brandt’s voles, and indicate that the impact of photoperiod in adult voles can be flexible in vole adulthood, varying according to their natural life cycle. This suggests a bet-hedging survival strategy of photo-refractoriness with complex interactions between environmental cues and life history traits.

**Supplementary Information:**

The online version contains supplementary material available at 10.1186/s40851-025-00251-6.

## Introduction

Mammals living in the temperate zone utilize daylength (photoperiod) as a proximate cue in anticipating seasonal changes, a behavior known as photoperiodism, which allows them to optimize their energy budgets and reproduce during the most appropriate period [1]. In the early 1930s, Baker and Ranson (1932) first discovered that daylength is an environmental factor that influences the seasonal breeding of field voles (*Microtus agrestis*) [2]. Many small rodents, including hamsters and voles, have been determined to be photoperiodic through field surveys and indoor simulation experiments, making them ideal for researching photoperiodism and seasonal reproduction in mammals [3–6].

The critical photoperiod is a specific daylength that triggers reproductive activity in photoperiodic animals. In many rodent species, this is between 12 and 14 h of light per day [7–11]. Photoperiods longer or shorter than the critical photoperiod, are referred to as long- (LP) and short-(SP) photoperiods, respectively. Exposure to SP from birth can lead to delayed physical and sexual development [10, 12–15]. For example, the testicular mass of male Siberian hamsters (*Phodopus sungorus*) aged 60 days that were exposed to SP (8 h light per day) was one-tenth that of males kept under LP conditions [12]. Similarly, the testicular mass of marsh rice rats (*Oryzomys palustris*) gestated and reared to four weeks of age in SP (8–12 h light per day) was approximately 50 mg in size, which is less than a quarter of that in LP males [10]. Moreover, under SP conditions, spermatogenesis is mainly arrested at the primary spermatocyte stage, with a reduced number of primary spermatocytes and no tubular lumen formation [13, 16]. In many seasonal rodents, adults that experience a transition from LP to SP, exhibit testicular atrophy [17–19], decreased testicular mass, shortened seminiferous tubule diameters, and depletion of the seminiferous epithelium [20].

However, the inhibitory effect of SP on gonadal activity is not permanent. Testicular recrudescence spontaneously occurs when photoperiodic animals are exposed to long-term SP conditions [16, 21]. For example, the atrophic testicles of male Siberian and Syrian hamsters (*Mesocricetus auratus*) regrow to an LP-like state, and their reproductive system is reactivated after 10–20 weeks [22, 23]. This phenomenon, known as photo-refractoriness, may be due to depletion of the pineal gland or desensitization of its target tissues [24, 25]. This loss of responsiveness to short daylength can be beneficial for LP breeders in preparing for reproduction before spring [26]. In rodents, photo-refractoriness appears only to occur under SP exposure, while it has been reported in some avian species exposed to LP conditions. For instance, testicular regression was found in adult male European starlings (*Sturnus vulgaris*) housed in long-term LP exposure conditions [27]. Such LP refractoriness allows birds to reduce the size of their reproductive organs and energy expenditure when they are not needed and ends the breeding season [28]. However, this phenomenon is rarely reported in rodents.

Brandt’s vole (*Lasiopodomys brandtii*) is a small, herbivorous rodent with a short lifespan (less than 14 months), mainly inhabiting the steppes of the Mongolian Plateau, in regions including China, Mongolia, and Russia [29, 30]. To adapt to the high latitude and seasonally changing environment, wild voles have to breed seasonally. Their breeding season usually begins in early spring (early March) and lasts until autumn (late August; [31, 32]). During the summer, the population size reaches its peak [33]. Moreover, the annual day length, gonadal mass, and expression of the hypothalamic deiodinase 2 (*Dio2*) gene, which is considered the transmitter of photoperiodic signals, all peak around the summer solstice [32]. Analysis of fecal testosterone levels of voles living in semi-natural enclosures revealed that males born in spring had faster gonadal development than those born in summer or autumn [34]. This suggests that there should be one or more regulatory factors to adjust the patterns of gonadal development of voles born in different seasons. The findings from these previous studies suggest that the most likely factor is photoperiod.

The aim of the present study was to investigate the impact of photoperiod on postnatal testicular development in Brandt’s voles. We hypothesized that (i) testicular development in young male voles born under LP conditions would be faster than that of young male voles born under SP conditions and, (ii) that prolonged exposure to a constant photoperiod could result in photo-refractoriness, i.e., spontaneous reversal of testicular activity. We divided pregnant voles into two groups, exposed to LP and SP, respectively. We measured the body mass and testicular size of male offspring weekly from postnatal 3 weeks (PNW3) to PNW19. We also collected testicular mass from the day of birth to compare their gonadal activity and histological features of the seminiferous epithelium. Our results showed that SP completely inhibited postnatal testicular development of Brandt’s voles until they were 10 weeks old, after which photo-refractoriness occurred. Interestingly, photo-refractoriness occurred not only under SP conditions, but also under LP exposure, with half of the males being refractory. This suggests that Brandt’s voles adopt a bet-hedging survival strategy to ensure the existence of the population in various and unpredictable seasonal shifts.

## Materials and methods

### Animals and housing conditions

Healthy parent voles were randomly selected from a laboratory breeding colony over a period of 10 years and 20 generations. The voles were kept in plastic cages (29 × 17 × 12 cm) containing wood shavings as substrate. The environment was maintained at an LP condition with a cycle of 16 h of light and 8 h of darkness (16L:8D), with the lights turning on at 06:00 am. The temperature was kept at 23 °C ± 2 °C. Food (standard rabbit feed) and water were provided *ad libitum*, and the nesting materials were renewed every three weeks. All procedures conformed to the institutional guidelines for animal use and care of the Institute of Plant Protection at the Chinese Academy of Agricultural Sciences (Protocol No. Ipp−202005R003).

### Photoperiod regimes and physiological measurements

A mating group consisting of one male and two females was kept together for 10 days. Afterward, the animals were isolated. One female was kept in the original LP cycle, while the other was shifted to an SP cycle of 8 h of light and 16 h of darkness (8L:16D). The lights were turned on at 08:00 am. These offspring were weaned at postnatal 3 weeks (PNW3), and 3–5 male siblings were housed together in a single cage.

**Fig. 1:**
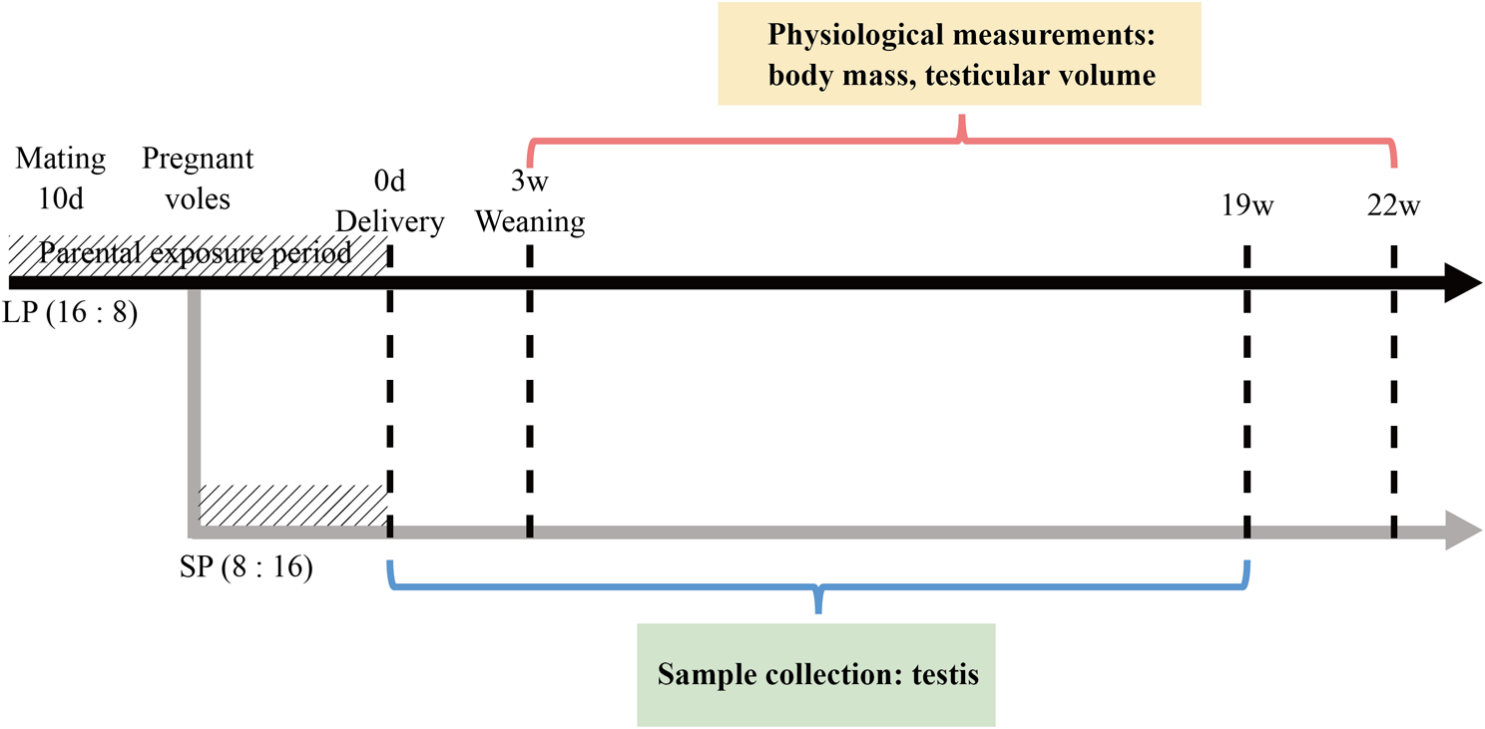
Experimental design. The black solid arrow indicates the duration of an LP (long photoperiod) group, and the grey solid arrow shows the duration of an SP (short photoperiod) group. Birth, weaning, and the end of the experiment are marked with dotted lines. The red bracket shows the period for which physiological measurements were taken from postnatal 3–22 weeks, and the blue bracket indicates sample collection from postnatal 0 day to 19 weeks. The hatched area represents the parental exposure period. Parental voles were maintained under long photoperiod conditions, and after 10 days of pairing, half of the pregnant females were transferred to short photoperiod conditions for delivery

Sixteen LP and fifteen SP males from different colonies were implanted with animal microchips (RBC-Z00, 1.25 × 7 mm, Beijing Raybaca Technologies Co., Ltd., China) for individual identification. Body mass was measured weekly from PNW3, and the left testicular volume was measured from PNW4 to PNW22 (Fig. 1). Additionally, the body mass and testicular volume of nineteen LP and twenty SP young males were monitored from PNW8 until they were sacrificed at PNW19. The testes were measured using digital vernier callipers, and the estimated testis volumes were calculated as the product of width^2^ × length [22]. Based on the current data, the testicular volume with complete spermatogenic cycles should be greater than 400 mm^3^, which was defined as the minimum criterion of sexual maturity for male voles.

At PNW19, SP voles with functional testes significantly exceeding the criterion volume were defined as a subgroup called “SP photorefractory (SP-PR)”, while LP males with atrophic testes below 400 mm^3^ were defined as a subgroup called “LP photorefractory (LP-PR)”. The other males with their original testicular size in the two groups were defined as “LP-response (LP-R)” and “SP-response (SP-R)”, respectively.

### Sample collection

Two to twenty male voles were sacrificed from birth (postnatal day 0, PND 0) to PNW19 for dissection (see Supplementary Table 1). Following ether anaesthesia, the voles were euthanized, and their bilateral testes were removed and weighed. The left testes were fixed in 10% neutral formalin at 4 °C for sectioning and staining.

### Testicular sections and haematoxylin & Eosin (H&E) staining

Fixed testes were processed by dehydration, permeation, wax-dipping, and embedding in paraffin and then cut into 4 μm thick sections. Afterwards, the slices were dewaxed with xylene (in three 8 min cycles) and hydrated by serial immersion in 100%, 90%, 80%, and 60% ethanol (all for 8 min cycles).

The testicular sections were examined using standard haematoxylin and eosin (H&E) staining (Soonbio, Beijing, China). After staining with haematoxylin for 4 min and rinsing with running water for 5 min, the sections were soaked in hydrochloric acid for 3 s, followed by 10 min of rinsing with running water and 20 s of immersion in 0.5% ammonia. The sections were then rinsed with running water for 10 min, stained with eosin for 1 min, and briefly immersed in distilled water. Finally, the sections were dehydrated in a series of graded ethanol concentrations (80%, 95%, and 100%), cleared in xylene and mounted with neutral gum.

### Histological analysis

To evaluate the activity of testes, the tubular diameters of 50 transverse sections of seminiferous tubules were randomly measured from one piece of slices for each animal, while the area of the cell nucleus and cytoplasm of 10 Leydig cells were calculated to determine the nucelo-cytoplasmic ratio, which reflects the capacity for testosterone production [35].

To evaluate the development of the seminiferous tubules from birth to adulthood (PNW10), the proportions of each tubular type were counted in a single testicle slice of each vole using light microscopy and the Image Analysis System 11 application (Beijing Changheng Rongchuang Technology Co., Ltd.). Based on the leading germ cells, i.e., the most advanced type of germ cells in the seminiferous epithelium, the tubules were classified into five stages: (i) pre-meiosis, consisting only of Sertoli cells and spermatogonia in the mitosis stage; (ii) leptotene or zygotene spermatocytes (l/z-spc), in the meiosis I stage with small primary spermatocytes occurring; (iii) pachytene spermatocytes (P-spc), in the meiosis I stage with large primary spermatocytes; (iv) round spermatids (R-spt), following secondary meiosis with small round spermatids; and (v) elongate spermatids (E-spt), observed as spermatids with tails [36]. Approximately 100 random tubular cross-sections were chosen from each slice to calculate the number of different stages.

To evaluate the degree of tubular degeneration during photo-refractoriness at PNW19, 30 tubular cross-sections were randomly chosen from each slice. According to the standards of Seco-Rovira et al. (2015) [20], the degree of tubular degeneration was categorized into three stages: (i) mild regression (MR), characterized by a decrease in the development of elongate spermatids in the lumen of the seminiferous tubule and a thinning of the germinal epithelium; (ii) strong regression (SR), where the germinal epithelium regressed until, but not beyond, the appearance of round spermatids in the lumen of the seminiferous tubules; and (iii) total regression (TR), in which the germinal epithelium regressed only until spermatocytes were observed and no round spermatids could be observed.

### Statistical analysis

All the data were analysed using the *SPSS 19 package*. Group differences were examined using the independent-sample *t* test. Data at different postnatal ages were evaluated by one-way ANOVA followed by least significant difference (LSD) tests. Two-way ANOVA was used to determine the interaction between photoperiod and postnatal time. Paired *t* tests were utilized to detect the significance of changes in testicular volume from PNW10 to PNW19. Pearson’s chi-square test was used to determine the differences in the proportion of tubular states. Differences were considered significant at *P* < 0.05.

## Results

### Somatic and testicular development of male voles

#### Body mass

**Fig. 2:**
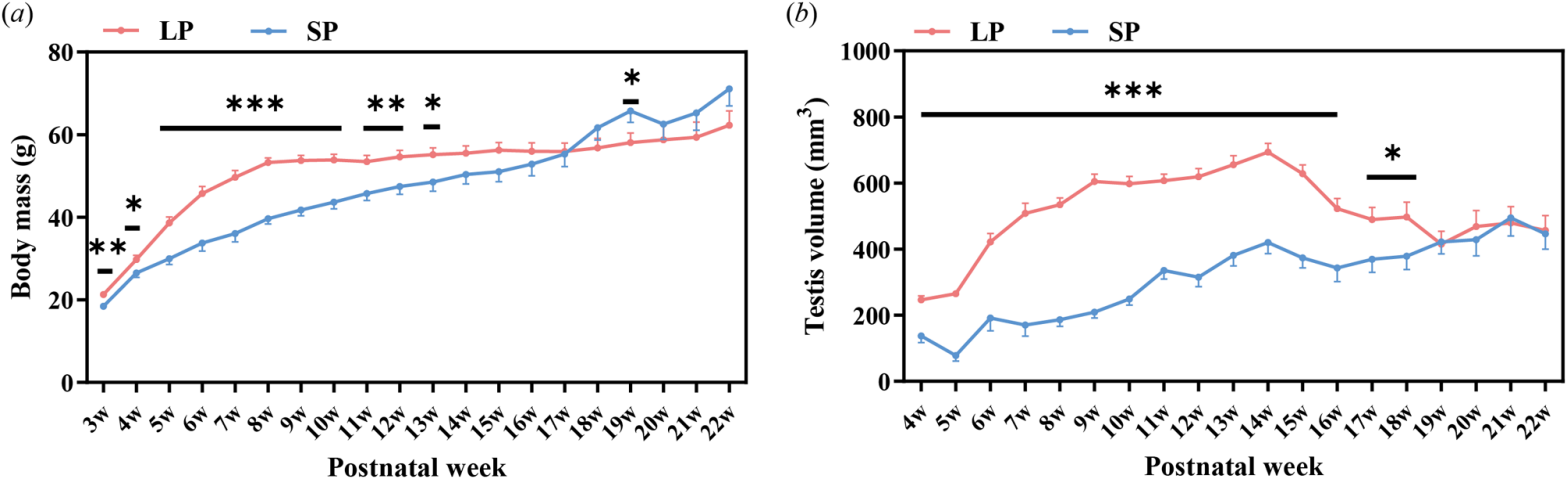
Postnatal development of body mass (***a***) and testis volume (***b***) in male Brandt’s voles under long and short photoperiods. Data were analysed using the independent-sample *t* test. Asterisks (*) indicate significant differences between LP and SP; *: *P* < 0.05, **: *P* < 0.01, ***: *P* < 0.001. Data are shown as the mean ± SEM. LP, long photoperiod; SP, short photoperiod; w, postnatal week

From weaning at PNW3, LP males had significantly heavier body mass than SP group (independent-sample *t* test: *t* = 3.169, *P* = 0.004), which was maintained until PNW13 (all *P* < 0.05; Fig. 2a and Supplementary Table 2). Within 20 weeks from PNW3 to PNW22, both the LP and SP groups significantly increased their body mass (one-way ANOVA: LP, *F* = 17.188, *P* < 0.001; SP, *F* = 22.166, *P* < 0.001) but displayed different growth patterns. LP males significantly gained 149.6% of their initial mean body mass from 21.3 ± 2.0 g at PNW3 to 53.3 ± 6.4 g at PNW8 (one-way ANOVA: *F* = 94.273, *P* < 0.001) and then remained stable from PNW8 (one-way ANOVA: *F* = 1.209, *P* = 0.265). While SP males kept a slow but stable somatic growth at approximately 2.6 g per week and caught up with LP males at PNW17. The SP males even significantly surpassed LP group in body mass at PNW19 (*t* = -2.116, *P* = 0.038). Two-way ANOVA indicated a significantly heavier body mass in LP compared to SP (*F* = 27.809, *df* = 1, *P* < 0.001; Fig. 2a), with photoperiodic effects (*F* = 4.547, *df* = 19, *P* < 0.001) in whole 20 weeks.

#### Testicular volume

Compared with SP males, LP males had significantly larger testicular sizes from PNW4 to PNW18 (all *P* < 0.05; Fig. 2b and Supplementary Table 3). The testicular volume of both groups displayed significant changes throughout the entire period (one-way ANOVA: LP, *F* = 14.261, *P* < 0.001; SP, *F* = 8.215, *P* < 0.001). The testicular size of LP males displayed a rapid increase from PNW4 to PNW9 and then a slower growth until reaching its peak at PNW14 (706.71 ± 136.26 mm^3^, one-way ANOVA: *F* = 39.506, *P* < 0.001). After that, there was a sharp 40% decrease in the following five weeks until PNW19 (one-way ANOVA: *F* = 6.782, *P* < 0.001), after which the size remained stable. However, similar to body mass, the testicular size of SP males continued to slowly increase until reaching its peak at PNW21, with an average of 12.34% weekly. At PNW19, both groups had similar testicular sizes. Overall, LP males had a significantly larger testicular size than SP males (two-way ANOVA: *F* = 321.858, *df* = 1, *P* < 0.001), and a significant photoperiodic effect was detected (*F* = 7.703, *df* = 18, *P* < 0.001).

#### Testis mass and seminiferous tubule diameter

**Fig. 3:**
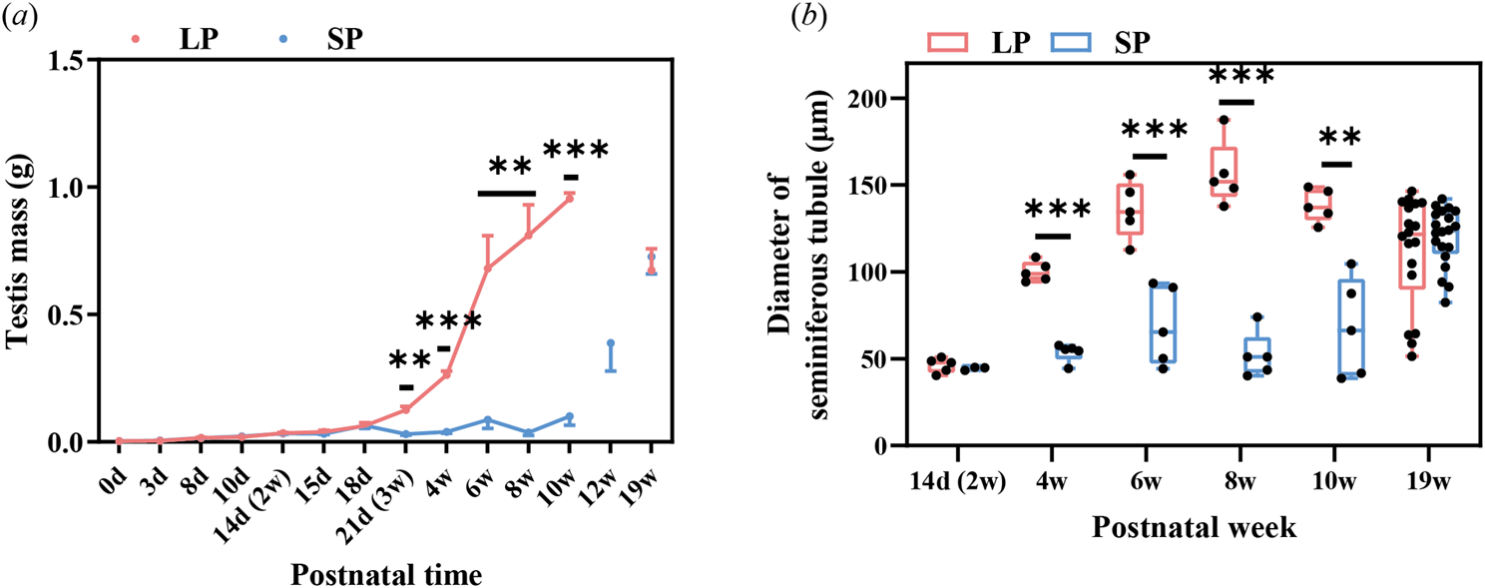
Postnatal development of testis mass (***a***) and diameters of seminiferous tubules (***b***) obtained after dissection in male Brandt’s voles under long and short photoperiods. Data were analysed using the independent-sample *t* test. Asterisks (*) indicate significant differences between LP and SP; *: *P* < 0.05, **: *P* < 0.01, ***: *P* < 0.001. Data are shown as the mean ± SEM. LP, long photoperiod; SP, short photoperiod; d, postnatal day; w, postnatal week

Both groups had similar bilateral testicular mass from birth to PND18 (Fig. 3a). However, three days later, the testes mass of LP males significantly exceeded that of the SP group until PNW10 (PNW3: *t* = 4.038, *P* = 0.010; PNW4: *t* = 13.667, *P* < 0.001; PNW6: *t* = 4.479, *P* = 0.002; PNW8: *t* = 6.465, *P* = 0.003; PNW10: *t* = 20.300, *P* < 0.001). During this period, LP males gained 14.9 times the testes mass (954.3 ± 73.1 mg), but the SP group only increased 1.6 times (100.4 ± 111.1 mg). Nevertheless, in the following two weeks, the testes of SP males rapidly grew 4 times (387.6 ± 244.8 mg). At PNW19, LP and SP males had the same testes mass again, while LP males lost 29.4% of their mass, but SP males grew 7 times compared to PNW10.

LP and SP had similar diameters of seminiferous tubules (~ 45 μm) at PNW2 (Fig. 3b). From PNW4 to PNW10, SP males had significantly shorter tubular diameters than LP males (PNW4: *t* = 13.426, *P* < 0.001; PNW6: *t* = 5.329, *P* < 0.001; PNW8: *t* = 10.206, *P* < 0.001; PNW10: *t* = 5.241, *P* = 0.004). At PNW19, they had similar tubular diameters. From PNW2 to PNW19, both LP and SP males displayed significant fluctuations in the diameters of the seminiferous tubules (one-way ANOVA: LP, *F* = 14.112, *P* < 0.001; SP, *F* = 27.285, *P* < 0.001). A significant increase in the diameters of the seminiferous tubules occurred before PNW6 in LP males (LSD: PNW4 vs. PNW2: *P* < 0.001; PNW6 vs. PNW4: *P* = 0.021), but occurred after PNW10 in SP males. Similar to the testes mass at PNW19, compared to PNW10, the diameters of the seminiferous tubules in the LP group significantly decreased by 21.7% (LSD: *P* = 0.032), but significantly increased by 77.2% in the SP males (LSD: *P* < 0.001).

#### Differential development of testes in the photorefractory period

**Fig. 4:**
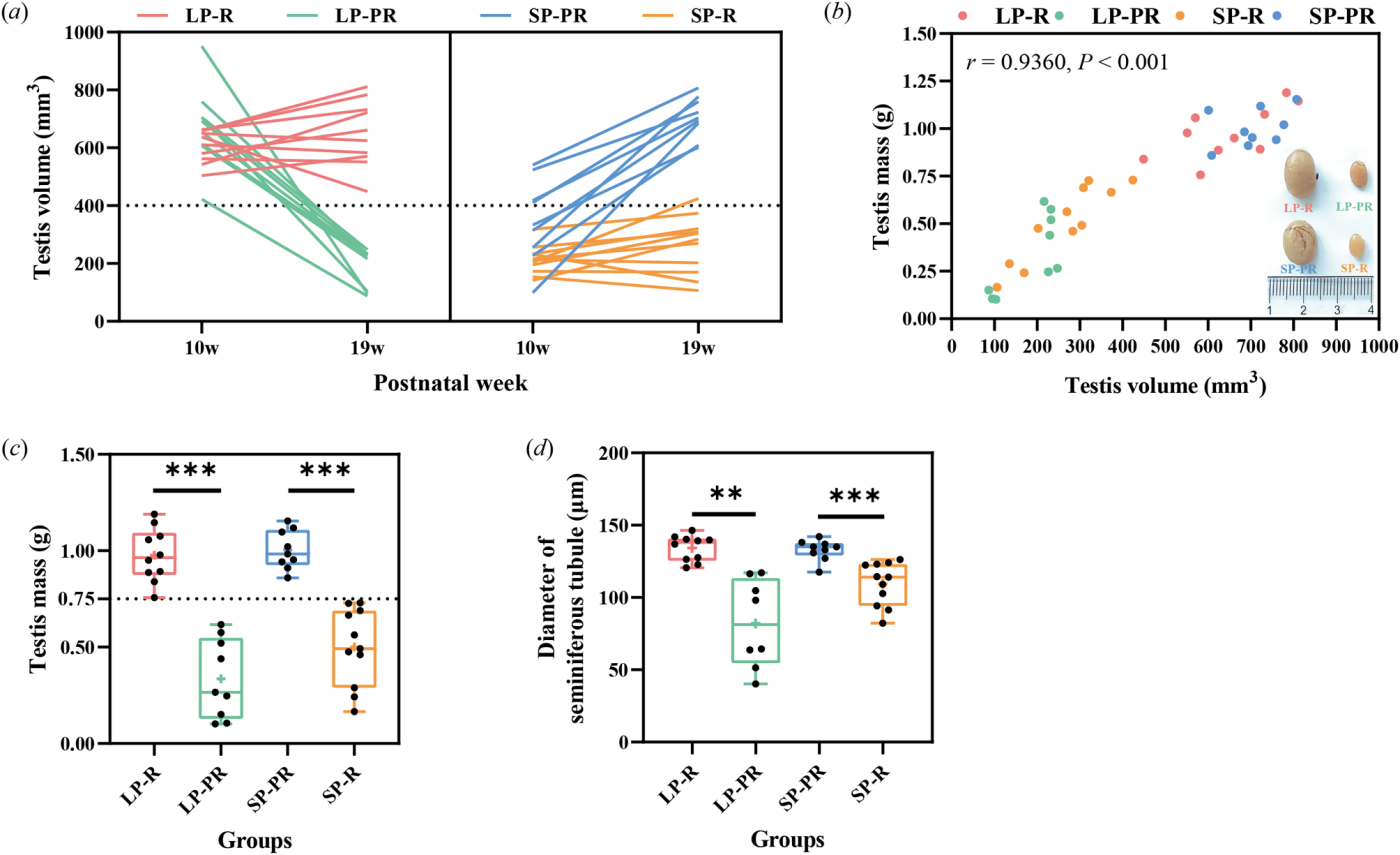
Testicular morphological differentiation of testes in Brandt’s voles that experienced long-term long and short photoperiodic exposure, including changes in testis volume from PNW10 to PNW19 (***a***), correlations between testicular volume and mass (***b***), testis mass (***c***) and diameters of seminiferous tubules (***d***) between the photo-responsive and photorefractory subgroups. Data were analysed using independent-sample *t* test. Asterisks (*) indicate significant differences; *: *P* < 0.05, **: *P* < 0.01, ***: *P* < 0.001. LP, long photoperiod; SP, short photoperiod; LP-R, males responding to long photoperiod with large testes; LP-PR, photorefractory males in long photoperiod with small testes; SP-PR, photorefractory males in short photoperiod with large testes; SP-R, males responding to short photoperiod with small testes

Comparing the testicular sizes of the same male between PNW10 and PNW19, the reversed tendencies occurred in either LP or SP group, respectively. Nine males in the LP (47.4%) and SP (45.0%) were classified into photorefractory groups, i.e., the “LP-PR” and “SP-PR” subgroups (Fig. 4a). The testis volume decreased by 72.6% (from 678.8 ± 140.7 mm^3^ to 185.7 ± 68.3 mm^3^; 95% Cl: 371.97–614.34 mm^3^, *t* = 9.384, *df* = 8, *P* < 0.001) in LP-PR males, while it significantly increased by 103.9% (from 346.6 ± 142.9 mm^3^ to 706.8 ± 70.3 mm^3^, 95% Cl: 263.30–457.04 mm^3^, *t* = 8.574, *df* = 8, *P* < 0.001) in SP-PR males. The other males in the two groups were classified into LP-R and SP-R groups, which kept active or inactive testes, respectively; no significant change was detected. Moreover, significantly positive correlations were found between testis volume and mass in all males (Pearson correlation: *r* = 0.9360 *P* < 0.001; Fig. 4b). According to the SP-R subgroup data, the simple linear regression equation was established as *y* (mass) = 0.001830*x* (volume) + 0.01797 (R^2^ = 0.8364, *P* < 0.001), and a testicular volume of 400 mm^3^ corresponded to a testicular mass of 0.75 g.

The medians of the testis mass in both LP and SP groups were close to 0.75 g at PNW19. Interestingly, it is also the dividing line between the photo-response and photorefractory groups (Fig. 4c). Compared with LP-R males, LP-PR males had significantly reduced testes mass (*t* = -8.063, *P* < 0.001; Fig. 4c) and tubular diameters (*t* = -4.679, *P* = 0.002; Fig. 4d). Reversed phenomena were found between SP-PR and SP-R (mass: *t* = -6.888, *P* < 0.001; diameter: *t* = -4.600, *P* < 0.001; Fig. 4c, d).

### Spermatogenesis under chronic long and short photoperiods

#### PND0 to PND18: Non-differential period

**Fig. 5:**
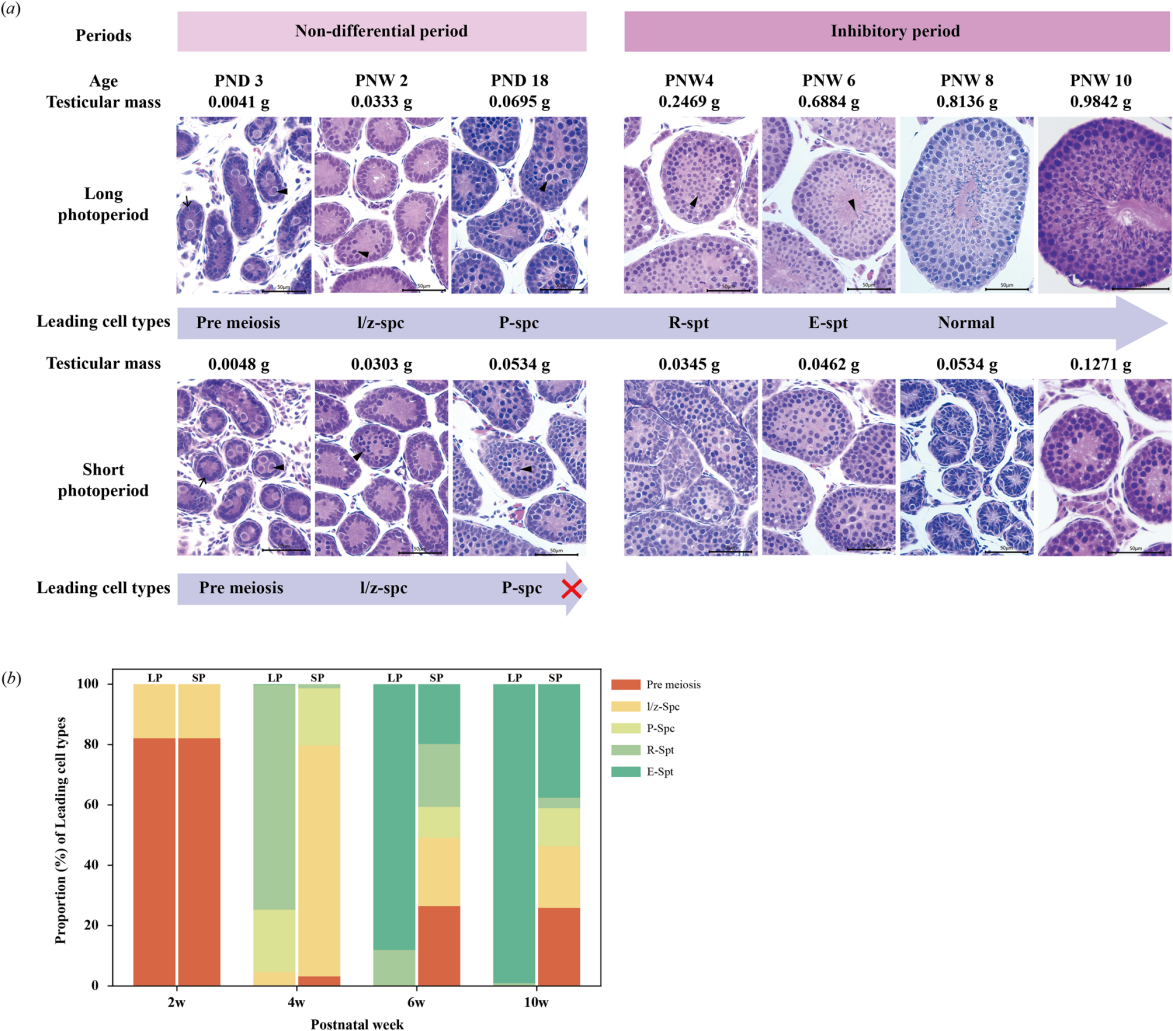
Testicular histology of Brandt’s voles under long and short photoperiods in the non-differential and inhibitory periods. Histological characteristics (***a***) and proportions (***b***) of seminiferous tubules in the mitosis phase (Pre-meiosis) and tubules with leading cells of leptotene or zygotene spermatocytes (l/z-spc), pachytene spermatocytes (P-spc), round spermatids (R-spt) and elongate spermatids (E-spt). In panel *a*, arrows indicate Sertoli cells (PND3), and arrowheads indicate different leading cells, including gonocytes (PND3), l/z-spc (PNW2), P-spc (PND18), R-spt (PNW4), and E-spt (PNW6). LP, long photoperiod; SP, short photoperiod. The scale bar represents 50 μm in 400×

The differentiation of germ cells was similar between LP and SP males before PND18. At PND3, the seminiferous tubules were lined with central gonocytes with large and round nuclei and Sertoli cells near the basement membrane and oval to elongated nuclei (Fig. 5a). At PND14 (PNW2), the Sertoli cells stopped dividing, and spermatocyte development (meiosis) began, accompanied by adluminal l/z-spc with condensed chromatin, clear cytoplasm, and distinct cell borders. Approximately 20% of the tubular sections contained l/z-spc in both groups (Fig. 5b). By PND18, P-spc appeared, with less densely packed nuclear chromatin than l/z-spc.

#### Short-photoperiod inhibitory period: PNW3 to PNW10

At PNW4, the proportion of seminiferous tubules with R-spt was 75% in the LP group but was only 1% in the SP group (Fig. 5b). Despite most tubules in SP testes producing primary spermatocytes, the number of spermatocytes was still insufficient. At PNW6, 88% of tubular cross-sections of LP males were undergoing complete spermatogenesis, whereas each of the five types of tubular cross-sections in the SP group accounted for a certain proportion, indicating unsynchronized development. By PNW10, 99% of the tubular sections in LP testes exhibited normal spermatogenesis, but 26% of the tubular cross-sections were arrested at the pre-meiosis stage in the SP group. Notably, 38% of the tubular cross-sections exhibited normal spermatogenesis.

#### Photorefractory period: after PNW10

**Fig. 6:**
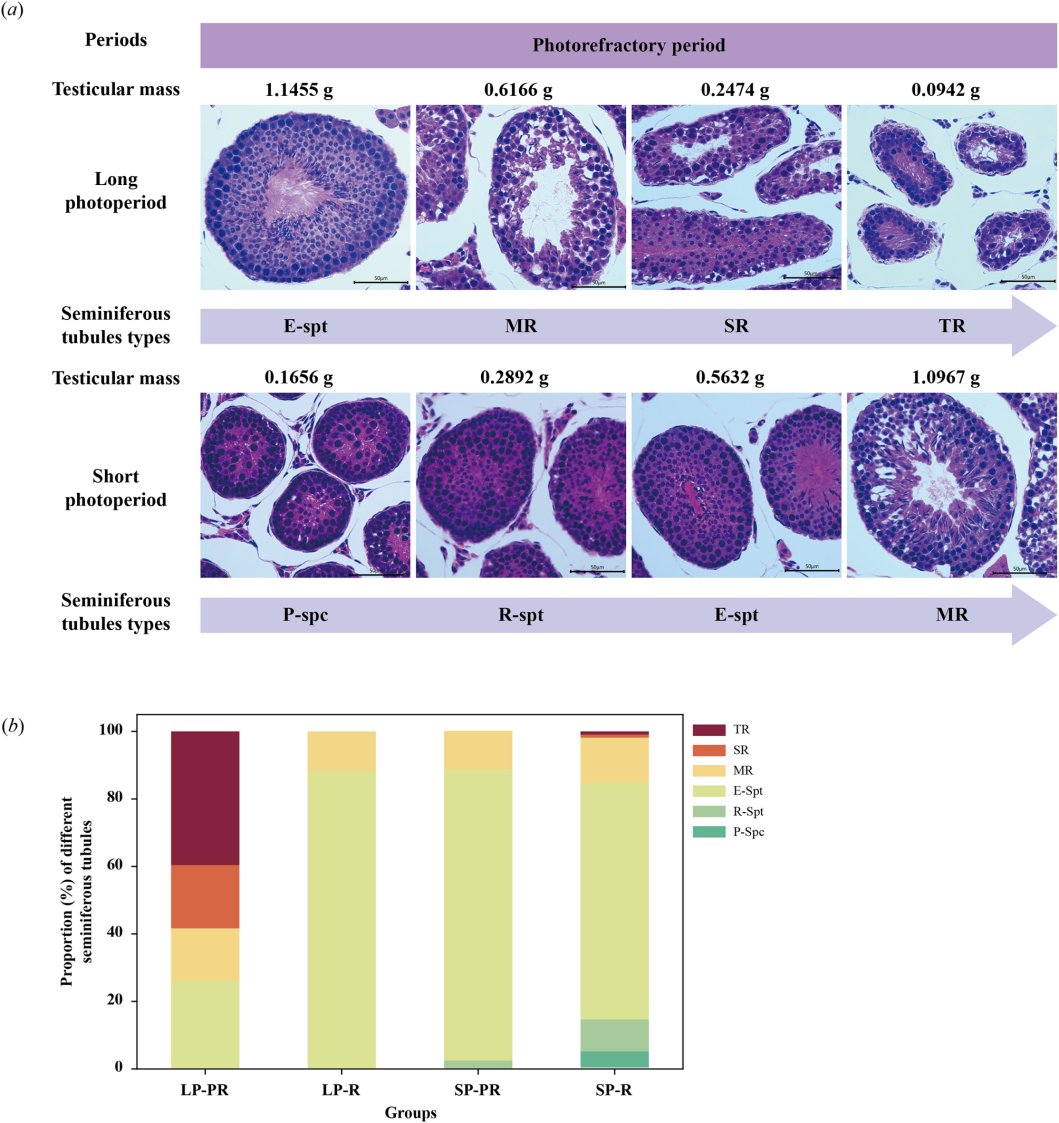
Testicular histology of Brandt’s voles under long and short photoperiods in the photorefractory period. Histological characteristics (***a***) and proportions (***b***) of mildly regressed (MR), strongly regressed (SR), total regressed (TR) seminiferous tubules and tubules with leading cells of pachytene spermatocytes (P-spc), round spermatids (R-spt) and elongate spermatids (E-spt). LP, long photoperiod; SP, short photoperiod; LP-R, males respond to long photoperiod with large testes; LP-PR, photorefractory males in long photoperiod with small testes; SP-PR, photorefractory males in short photoperiod with large testes; SP-R, males respond to short photoperiod with small testes. The scale bar represents 50 μm in 400×

At PNW19, LP males displayed various degrees of regression in the spermatogenesis (Fig. 6a). The mildly regressed (MR) tubular cross-sections showed a decrease in late spermatid development in the lumen of the seminiferous tubules and a thinning germinal epithelium. The strongly regressed (SR) sections demonstrated an arrest in round spermatids, with almost no elongated spermatids. When only spermatocytes were visible, the tubules had entered total regression (TR), with the basement membrane curling and thickening. However, SP males were in the process of testicular development, and spermatogenesis status were in the emergence of P-spc, R-spt, and E-spt from small to large testes (Fig. 6a).

In both the LP-R and SP-PR groups, nearly 90% of the seminiferous tubules were in the E-spt stage. Compared with the LP-R group, the LP-PR group showed a dramatic decrease in the proportion of E-spt tubular sections (88% in LP-R to 26% in LP-PR), while the percentage of regressed tubular sections significantly increased (12% to 74%; Pearson χ^2^ = 215.510, *P* < 0.001), as well as 40% sections in the TR stage. Additionally, the SP-R group had more developing tubules, such as P-spc (5%) and R-spt (9%), as well as a small number of regressed tubules, including MR (1%) and TR (1%; Fig. 6b).

#### Nucelo-cytoplasmic ratio of testicular Leydig cells under long and short photoperiod

**Fig. 7:**
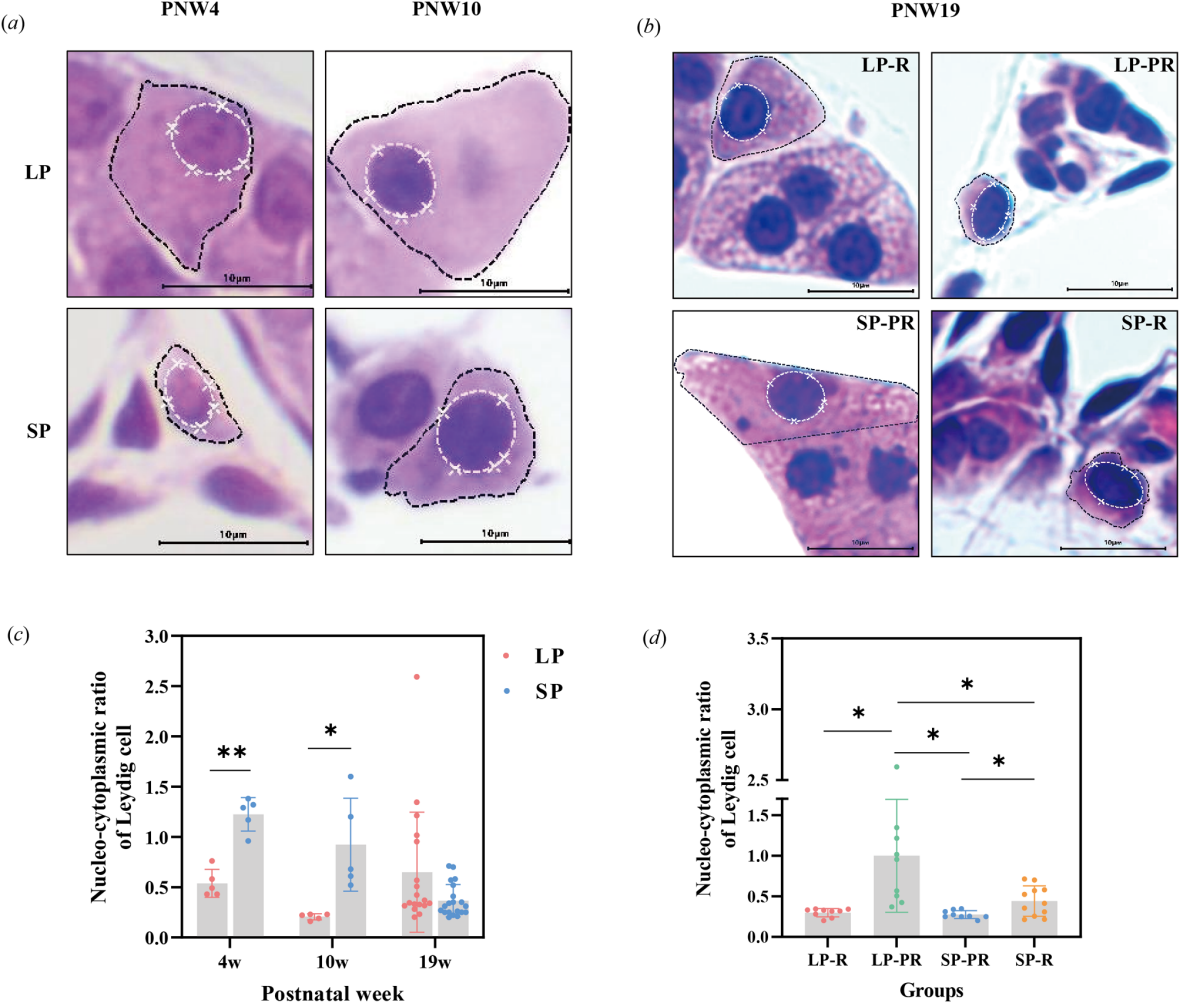
Morphology (***a***, ***b***) and nucleo-cytoplasmic ratio of Leydig cells in Brandt’s voles between long and short photoperiods at PNW4, PNW10, and PNW19 (***c***) and between four subgroups at PNW19 (***d***). The black dotted line surrounds the total area of a single Leydig cell, and the white dotted line surrounds the area of the nucleus (***a***, ***b***). The data were analysed using the independent-sample *t* test. Asterisks (*) indicate significant differences between LP and SP (***a***) or between any two of the four subgroups at PNW19 (*b*); *: *P* < 0.05, **: *P* < 0.01. LP, long photoperiod; SP, short photoperiod; LP-R, males respond to long photoperiod with large testes; LP-PR, photorefractory males in long photoperiod with small testes; SP-PR, photorefractory males in short photoperiod with large testes; SP-R, males respond to short photoperiod with small testes; w, postnatal week. The scale bar represents 10 μm in 400×

The Leydig cells in the LP testes were either isolated or clustered in the interstitium at PNW4 and PNW10, while the interstitial tissue in the SP group showed developmental retardation with a mass of undeveloped Leydig cells (Fig. 7a). As development progressed, the cytoplasm of LP Leydig cells grew normally, and the nucleo-cytoplasmic ratio decreased from 0.54 ± 0.14 at PNW4 to 0.21 ± 0.03 at PNW10, a reduction of half (Fig. 7c). In comparison, the nucleo-cytoplasmic ratio of the SP group decreased from 1.22 ± 0.17 at PNW4 to 0.92 ± 0.46 at PNW10. Overall, the interstitial tissue in LP males developed faster than that in the SP group until PNW10 (PNW4: *t* = -7.091, *P* < 0.001; PNW10: *t* = -3.456, *P* = 0.026). By PNW19, there was no significant difference between the two groups (*t* = 1.947, *P* = 0.066), but the mean ratio in LP males tripled compared to that in PNW10 (Fig. 7c). In contrast, the mean ratio in the SP group decreased by 60.4%, reaching 0.37 ± 0.16 (Fig. 7c). Additionally, compared with the LP-PR and SP-R males, the LP-R and SP-PR males had fully developed Leydig cells with a larger cytoplasmic area (Fig. 7b). Furthermore, the LP-PR males had a higher nucleo-cytoplasmic ratio than the LP-R subgroup (*t* = 2.824, *P* = 0.025), and the SP-R males had a higher ratio than the SP-PR subgroup (*t* = 2.837, *P* = 0.015). Notably, the nucleo-cytoplasmic ratio of LP-PR was significantly higher than that of SP-R (*t* = 2.338, *P* = 0.049), indicating different stages of small testes under long and short photoperiods (Fig. 7d).

## Discussion

In this study, we investigated the effects of long (LP) and short (SP) photoperiods on male Brandt’s voles from pregnancy to postnatal week 22. We found that SP significantly suppressed somatic and gonadal development in male voles from weaning to adulthood but not from birth. Furthermore, photorefractoriness was observed after postnatal week 10 in both photoperiodic groups. Interestingly, not only SP males but also LP ones reversed their gonadal activity status, with the ratio being similar in both groups. Our findings demonstrate that photoperiod or daylength plays a role in determining developmental and seasonal breeding patterns in Brandt’s vole.

### Somatic growth was fast in LP, but slow in SP

Our results showed that SP had a significant impact on the somatic growth of Brandt’s voles from weaning to adulthood. After a quick increase in body mass for five weeks following weaning, LP males maintained a consistent body mass of approximately 54 g for 13 weeks. In contrast, SP males had a lower body mass than LP males until PNW14, indicating a developmental delay of at least 6 weeks compared to LP males. However, SP males eventually caught up and even exceeded the body mass of LP males by PNW19. Body mass is an important indicator of the nutritional status of animals, and changes in body mass are influenced by the balance between energy intake and expenditure [37–39]. The mechanism of energy balance following a change in photoperiod appears to vary among small rodents. For example, Siberian hamsters experience body mass loss under SP conditions due to a decrease in food intake [40, 41], while Djungarian hamsters (*Phodopus sungorus*) experience a decrease in body mass under SP conditions primarily due to a reduction in adipose tissue mass [42]. Previous studies on Brandt’s voles have shown that SP-induced body mass loss is due to an increase in energy expenditure rather than a decrease in food intake [43, 44]. The delayed somatic development of voles may be attributed to the increased energy consumption under SP conditions. In contrast, the collared lemmings (*Dicrostonyx groenlandicus*) exposed to SP increased in body mass without increasing food intake or digestive efficiency, likely due to a decrease in resting energy expenditure [45, 46]. Our study revealed that SP males caught up with LP males in body mass after PNW10 and surpassed them by PNW19, possibly accompanied by a decrease in metabolic rate.

### Overwhelming SP-inhibition of testicular development until adulthood

Our results revealed that there was no significant difference in testicular development between the LP and SP groups from birth (PND0) to weaning (PNW3), which is referred to as “non-differential stage”. This is consistent with previous research on Djungarian hamster, which showed a similar increase in testis mass under both LP (16L:8D) and SP (10L:14D) conditions at PND15 [12]. Short photoperiod did not inhibit the initiation of spermatogenesis, but rather the production of primary spermatocytes. Approximately 20% of tubular sections contained leading cells with leptotene or zygotene spermatocytes, indicating that both LP and SP males began meiosis in the first wave of spermatogenesis at two weeks of age. This may be attributable to the development of the pineal melatonin rhythm, which typically occurs between postnatal days 15 and 20 in Syrian and Siberian hamster, respectively [47]. The circadian rhythm of melatonin produced by the pineal gland plays a crucial role in translating information about day length and influencing the hypothalamus-pituitary-gonadal axis [48, 49]. Studies have shown that the onset of the pineal melatonin rhythm is closely linked to the ability of SP to inhibit the peak in serum follicle-stimulating hormone (FSH) levels and hider gonadal growth in Siberian hamster by 20 days of age [12]. This suggests that the maturation of a pineal-dependent mechanism is essential for the photoperiodic regulation of gonadal development in hamsters and voles.

During the SP-inhibitory stage from PNW3 to PNW10, SP Brandt’s voles experienced a significant delay in testicular development, including size, mass, and spermatogenesis. At PNW10, the testicular size and mass of the more developing SP males were similar to LP males at PNW4. Histological analysis showed that spermatogenesis began at PNW6 with an increase in tubular diameter and the percentage of tubules containing E-spt, but mature sperm did not form until PNW10. These findings support the inhibitory effects of SP on gonadal development in male voles. Previous studies have also documented the inhibitory effect of SP on gonadal development in other photoperiodic small rodents, such as montane vole (*Microtus montanus*) [50, 51], Djungarian hamsters [12, 52], marsh rice rat (*Oryzomys palustris*) [10], white-footed mice (*Peromyscus leucopus*) [53] and meadow voles (*Microtus pennsylvanicus*) [54, 55]. Previous studies in Brandt’s voles have shown that LP males had larger testes than SP males at PND60 or PNW12 [56, 57]. However, there is a lack of literature on the histological characteristics in developmental processes during SP inhibitory conditions. Our study confirmed that SP inhibits testis development in young male voles, leading to a delay in puberty. LP males had R-spt in the seminiferous epithelium at PNW4 and entered the normal cycle by PNW10, while SP males showed a blockage in P-spt production until PNW10. Similarly, Djungarian hamsters born under SP conditions also exhibited this phenomenon, with spermatogenesis being halted primarily at the mid-pachytene stage from PND22 to PND64. During this time, no tubular lumen was formed, and there was a decrease in the number of preleptotene spermatocytes [13]. These results suggest that maternal photoperiod experience can influence the gonadal development of offspring after weaning.

The nucelo-cytoplasmic ratio of Leydig cells in SP male voles was found to be significantly higher compared to those in LP males from PNW4 to PNW10. Leydig cells play a crucial role in testosterone synthesis within the testicular interstitium [58]. Exposure to SP was shown to decrease the cytoplasmic volume of Leydig cells, particularly affecting the saccular variety of the endoplasmic reticulum and weakening testosterone synthesis [59]. Though testosterone levels were not measured in this study, the higher nucelo-cytoplasmic ratio in Leydig cells of SP males indicates suppressed testosterone synthesis, which likely contributes to the inhibited testicular development observed in these males. These findings suggest that the inhibition of testicular mass gain in SP male voles can be attributed to changes in morphometric parameters, such as tubular diameter and nucelo-cytoplasmic ratio of Leydig cells, resulting from blocked spermatogenesis, absence of late germ cells, and delayed development of Leydig cells.

### Photo-refractoriness of testes in chronic LP and SP exposure

In photoperiodic rodents, photo-refractoriness typically occurred after prolonged exposure to SP for more than 10 weeks, leading to spontaneous recrudescence of gonadal activity [60]. This phenomenon has been observed in various rodent species, such as white-footed mouse [53], Djungarian hamster [16], Siberian hamster [22] and Syrian hamster [23]. In the present study, we observed that male voles exposed to SP quickly developed their testes after PNW10 and experienced a significant increase in testes mass, reaching up to as much as 280% above 300mm^3^ in the following two weeks. Additionally, there was a revival of the meiotic process in the seminiferous tubules, accompanied by an enlargement of tubular diameter and a decrease in the nucelo-cytoplasmic ratio of Leydig cells. These findings are consistent with the spontaneous recrudescence of gonadal activity observed in adult Syrian hamster after 16 weeks of exposure to SP [21]. However, it is noteworthy that only approximately half of SP male voles in our study exhibited testicular recrudescence.

Interestingly, half of the LP males also experienced a decrease in testicular activity during the photorefractory stage. By PNW19, nine LP males had testes smaller than 400mm^3^, with the average testicular size and mass decreasing by 72.6% and 64.8%, respectively, compared to PNW10. Histological analysis revealed tubular regression from MR to TR in LP-PR males, with decreasing tubular diameters and an increase in the nucelo-cytoplasmic ratio of Leydig cells. It seems that LP-PR males naturally entered a non-reproductive state despite being under LP conditions. These data indicates an LP photo-refractory in Brandt’s vole, a phenomenon commonly observed in birds [61–64], but rarely reported in rodents, with the exception of collared lemmings (*Dicrostonyx groenlandicus*) [65, 66]. The histological characteristics of testicular degeneration in the LP-PR are similar to the SP degeneration observed in adult Syrian hamsters [20], both showing a progressive loss of germ cells from spermatids to spermatocytes.

Testicular regression can occur due to both aging and seasonal inhibition, but these processes have distinct outcomes. Aging-related regression is irreversible, while seasonal inhibition leads to reversible regression [67]. The reversibility of regression depends on the ability of spermatogonia to recover. In Syrian hamsters, exposure to SP condition results in a decrease in differentiated spermatogonia and an increase in undifferentiated spermatogonia [68]. In the present study, we observed degeneration of spermatids and spermatocytes in the testes of LP-PR males, but an increase in undifferentiated spermatogonia with large nuclei, indicating the potential for spermatogenesis restoration under suitable conditions. Further experiments are needed to test the proliferative activity and differentiation capacity of spermatogonia in regressed testes. Brandt’s voles have a life span of less than 14 months in the wild, but can live up to two years in laboratory housing [69]. Therefore, voles aged 19 weeks are not considered aged. Not all LP males showed LP-refractoriness, suggesting it is not an inevitable life history trait but a survival strategy for active overwintering. In our previous observations, testicular regression also occurred in adult voles from July to September in the wild population, indicating that adult voles born in the same year shut down their reproductive function before winter to conserve energy for overwintering.

### Potential neuroendocrine and ecological mechanisms for photoperiodism in Brandt’s vole

Seasonal breeders of mammals rely on the pineal gland to convert the photoperiod into melatonin, which plays a key role in regulating reproductive activity [70]. In conditions with longer period of melatonin secretion in SP conditions, the production of thyroid stimulating hormone (TSH) in the pars tuberalis (PT) is inhibited, leading to alterations in the dynamic expressions of *Dio2* and type III deiodinase (*Dio3*) enzymes in tanycytes [71, 72]. DIO2 converts thyroxine (T4) to bioactive triiodothyronine (T3), while DIO3 catabolizes both T4 and T3 to inactive metabolites, which results in a decrease of T3 level in the mediobasal hypothalamus [73], ultimately influencing reproductive function in rodents such as hamsters and voles. Studies have shown that maternal melatonin levels can affect fetal PT and regulate TSH levels, potentially starting before birth. For example, Siberian hamsters born in LP and SP conditions displayed significant differences in *Tshβ* mRNA expression in the PT and *Dio2* expression in the hypothalamus [15]. Additionally, transferring juvenile hamsters from LP to SP can rapidly increase *Dio3* mRNA expression in the hypothalamus within three days (PND18 to PND21) [18].

However, the exact neuroendocrine mechanisms underlying SP-refractoriness in rodents are not yet fully understood. In mammals, the hypothalamic-pituitary-gonadal axis stops response to the prolonged SP condition and becomes insensitive to a long duration of melatonin followed by an increase in FSH and luteinizing hormone [74]. In Siberian hamsters, the concentration of T_4_ in the hypothalamus plays a role in SP-refractoriness, as extended exposure to SP results in a decrease in T_4_-binding protein synthesis and a reduction in hypothalamic T_4_ uptake [22]. Milesi et al. (2017) reported that downregulation of *Dio3* is a common early event in activation of the gonadotropic axis during both LP exposure and SP-refractoriness in Syrian and Djungarian hamsters [75]. Our previous research also revealed that the seasonal dynamic expressions of hypothalamic *Dio2* and *Dio3* were closely linked to the annual photoperiodic cycle in wild vole populations, with the ratio of *Dio2* to *Dio3* peaking in June, suggesting a potential key role for these molecules in regulating seasonal breeding in Brandt’s voles [32]. Further investigation into the molecular pathways involved in overcoming suppression of SP could provide new insights for managing Brandt’s vole populations.

Photoperiodism in Brandt’s vole may play a crucial role in their fitness from an ecological perspective. Previous studies have shown that in seasonal breeding small rodents, rapid somatic and gonadal growth give those born in the early breeding season a better chance for reproductive success, while those born later in the season conserve energy for survival in the harsher autumn and winter [76, 77]. Photorefractoriness, the spontaneous reversal of gonadal activity, helps voles prepare for the upcoming breeding or non-breeding seasons [26, 78]. Interestingly, our study found that the voles with or without refractoriness were evenly differentiated in both LP and SP conditions, implying that Brandt’s voles adopt a “bet-hedging” strategy in photoperiodic phenotype on unpredictable environmental changes in the following season. The steppes of Inner Mongolia, the voles’ natural habitat, is in the temperate continental climate zone with unpredictable changes in seasonal shifts, such as dynamic fluctuations of ambient temperature and rainfall, which probably promote or delay the coming of spring and autumn [79]. It may be that voles with photoperiod-refractoriness bet on the advanced or normal seasonal shifts, such as warm spring and cold autumn, which could increase fitness by increased brood number in breeding season or survival in non-breeding season. This effect is reversed in photoperiod-response voles, which will be higher fitness in cold spring or warm autumn. This adaptability is likely a result of long-term evolutionary processes in response to the variable environment.

However, the origin of phenotypic differentiation among LP-R, LP-PR, SP-R, and SP-PR individuals remains unclear, whether it stems from epigenetic or genomic factors. LP and SP individuals derive from eight and nine distinct litters, respectively, with some litters containing both LP-R and LP-PR individuals, and others containing both SP-R and SP-PR individuals. Despite sharing a genetic background, phenotypic variations among littermates suggest that genetic factors may not be the primary determinants. Studies have demonstrated that epigenetic modifications in monozygotic twin pairs diverge with age, influenced by both external and internal factors [80]. Epigenetic influences are also plausible, as offspring exposed to the same external photoperiod conditions exhibit phenotypic differences despite similar environmental exposures. Experimental validation is necessary to confirm the presence of epigenetic modifications, such as differential methylation sites and histone acetylation patterns. It is hypothesized that this phenomenon may involve intricate neuroendocrine regulatory mechanisms. Given that photoperiodic signalling involves hierarchical processing through the hypothalamic-pituitary-gonadal axis, key regulatory nodes necessitate investigation, including melatonin responsiveness in the PT, hypothalamic sensitivity to TSH, T3-mediated regulation of GnRH neurons, and testicular responsiveness to gonadotropins. Further exploration of these pathways will offer crucial insights into the mechanistic underpinnings of photo-refractoriness in Brandt’s voles. Moreover, the distribution of Brandt’s voles has significantly contracted in recent years, with their southern range shifting several hundred kilometers north in China over the past two decades [81]. Therefore, elucidating the adaptive mechanisms to environmental changes in Brandt’s voles is imperative for comprehending their distribution dynamics and evolutionary history.

## Conclusion

The present study provides new insights into the effect of photoperiod on postnatal testicular development in Brandt’s vole. The inhibitory effect of short photoperiod appears after weaning. Young voles born in long photoperiod develop normally, while the puberty of young voles born in short photoperiod is delayed, mainly manifested by blocking the meiotic process in the seminiferous epithelium. However, long-term exposure to both long and short photoperiods results in photorefractoriness, which will be beneficial for the animals in facilitating active overwintering and initiating breeding before the onset of the following spring. In summary, photoperiod is a key environmental clue guiding the adaptation and seasonal reproduction of Brandt’s vole.

## Electronic supplementary material

Below is the link to the electronic supplementary material.

Supplementary Material 1: Table of sampling time and sample sizes of male Brandt’s voles throughout the experiment, showing the number of samples collected at each time point under long and short photoperiods. Note: “/” represents no sampling.

Supplementary Material 2: Body mass (mean ± SD) of male Brandt’s voles at different ages under long and short photoperiods. P value showing the effect of photoperiod and age on body mass. Independent-sample t test and one-way ANOVA were used for data analysis.

Supplementary Material 3: Testicular volume (mean ± SD) of male Brandt’s voles at different ages under long and short photoperiods. P value showing the effect of photoperiod and age on testicular volume. Independent-sample t test and one-way ANOVA were used for data analysis.

## Data Availability

The datasets used and analysed during the current study are available from the corresponding author on reasonable request.
